# One-year urinary and sexual outcome trajectories among prostate cancer patients treated by radical prostatectomy: a prospective study

**DOI:** 10.1186/s12894-021-00845-0

**Published:** 2021-05-17

**Authors:** Lin Yang, Jung Ae Lee, Emily Heer, Claire Pernar, Graham A. Colditz, Ratna Pakpahan, Kellie R. Imm, Eric H. Kim, Robert L. Grubb, Kathleen Y. Wolin, Adam S. Kibel, Siobhan Sutcliffe

**Affiliations:** 1 Department of Cancer Epidemiology and Prevention Research, Cancer Research and Analytics, Cancer Care Alberta, Alberta Health Services, 5th Floor, Holy Cross Centre, Box ACB, 2210 - 2 St. SW, Calgary, AB T2S 3C3 Canada; 2 Departments of Oncology and Community Health Sciences, University of Calgary, Calgary, Canada; 3 Division of Public Health Sciences, Department of Surgery, Washington University School of Medicine, 600 S. Taylor Ave., 2nd floor, Rm. 208S, Campus Box 8100, St. Louis, MO 63110 USA; 4 Agricultural Statistics Laboratory, University of Arkansas, Fayetteville, USA; 5 Department of Epidemiology, Harvard T.H. Chan School of Public Health, Boston, MA USA; 6 Division of Urological Surgery, Department of Surgery, Washington University School of Medicine, St Louis, USA; 7 Department of Urology, Medical University of South Carolina, Charleston, USA; 8 Coeus Health, Chicago, IL USA; 9 Division of Urology, Department of Surgery, Brigham and Women’s Hospital, Boston, USA; 10 Division of Health Behavior Research, Department of Preventive Medicine, Keck School of Medicine of the University of Southern California, 2001 N Soto St., Los Angeles, CA 90032 USA

**Keywords:** Localized prostate cancer, Benign prostatic hyperplasia, Radical prostatectomy, Urinary outcome, Sexual outcome

## Abstract

**Background:**

To examine one-year trajectories of urinary and sexual outcomes, and correlates of these trajectories, among prostate cancer patients treated by radical prostatectomy (RP).

**Methods:**

Study participants were recruited from 2011 to 2014 at two US institutions. Self-reported urinary and sexual outcomes were measured at baseline before surgery, and 5 weeks, 6 months and 12 months after surgery, using the modified Expanded Prostate Cancer Index Composite-50 (EPIC-50). Changes in EPIC-50 scores from baseline were categorized as improved (beyond baseline), maintained, or impaired (below baseline), using previously-reported minimum clinically important differences.

**Results:**

Of the 426 eligible participants who completed the baseline survey, 395 provided data on at least one EPIC-50 sub-scale at 5 weeks and 12 months, and were analyzed. Although all mean EPIC-50 scores declined markedly 5 weeks after surgery and then recovered to near (incontinence-related outcomes) or below (sexual outcomes) baseline levels by 12 months post-surgery, some men experienced improvement beyond their baseline levels on each sub-scale (3.3–51% depending on the sub-scale). Having benign prostatic hyperplasia (BPH) at baseline (prostate size ≥ 40 g; an International Prostate Symptom Index Score ≥ 8; or using BPH medications) was associated with post-surgical improvements in voiding dysfunction-related bother at 5 weeks (OR = 3.9, 95% CI: 2.1–7.2) and 12 months (OR = 3.3, 95% CI: 2.0–5.7); and in sexual bother at 5 weeks (OR = 5.7, 95% CI:1.7–19.3) and 12 months (OR = 3.0, 95% CI: 1.2–7.1).

**Conclusions:**

Our findings provide additional support for considering baseline BPH symptoms when selecting the best therapy for early-stage prostate cancer.

**Supplementary Information:**

The online version contains supplementary material available at 10.1186/s12894-021-00845-0.

## Background

Early-stage, localized prostate cancer can be managed in several ways, including watchful waiting, active surveillance, and curative modalities such as radical prostatectomy (RP), external radiotherapy, and brachytherapy. As each of these modalities has high survival rates [1], the benefits of curative therapies must be carefully weighed against their harms, including their side effects [2–4].

A number of studies have documented the natural history of prostate cancer treatment side effects [5–12]. For RP, in particular, studies indicate that men tend to experience an initial large decline in urinary and sexual function immediately after surgery (i.e., within the first two months), followed by a gradual improvement to near or below baseline levels by the first year post-surgery. One exception to this pattern is voiding dysfunction (or urinary irritation or obstruction). Symptoms of voiding dysfunction have been observed to improve beyond pre-surgical values in a few previous studies, presumably because of relief of urinary obstruction by prostatectomy in men with both prostate cancer and benign prostatic hyperplasia (BPH) [13]. These findings have led AUA, ASTRO, and SUO to recommend surgery over radiation therapy for patients with clinically localized prostate cancer and obstructive, non-cancer-related lower urinary tract dysfunction [14]. However, as this recommendation is Grade C, additional high-quality evidence from randomized controlled trials and prospective observational studies is still needed.

To our knowledge, voiding dysfunction is the only outcome in which improvement beyond pre-surgical values has been explored. Although mean improvement has not been observed in other outcomes, such as sexual function or bother, it is possible that examination of mean trajectories may have obscured improvement or other trajectories experienced by only a subset of participants—for instance, those who use medication and devices to treat their erectile dysfunction (ED) after surgery. Understanding the full range of side effect trajectories would be useful to help set patients’ expectations post-surgery and to further aid with treatment decision-making. Therefore, we analyzed data from the prospective Prostatectomy, Incontinence, and Erectile Dysfunction (PIE) Study to describe the full range of urinary and sexual side effect trajectories, the percentage of patients with improvement beyond baseline in each domain, and factors related to improvement in the first year post-surgery (i.e. BPH- and ED-related factors).

## Methods

### Study population and design

Prostate cancer patients were recruited into the PIE study from 2011 to 2014 at two sites, Washington University School of Medicine and Brigham & Women’s Hospital. All men undergoing RP for clinically localized prostate cancer were eligible, except for those who: (1) had previously undergone treatment for prostate cancer, radiation therapy to the pelvis (including bladder, rectum, or prostate), or major pelvic surgery (including penile implant or urinary sphincter); (2) had known urethral stricture or colostomy; (3) were unable to urinate and required indwelling catheterization; and (4) did not speak English. Men who received neo-adjuvant therapy or any additional prostate cancer-related therapies (e.g., radiation or hormonal therapies) during the one-year study follow-up were excluded from the analyses. The PIE study was approved by the institutional review boards at both institutions. All participants provided informed consent.

### Urinary and sexual outcomes

Patient-reported urinary and sexual outcomes were assessed at baseline before RP, and 5 weeks, 6 months, and 12 months post-RP by the modified Expanded Prostate Cancer Index Composite-50 (EPIC-50) [15]. This validated scale includes sub-scales for urinary function (i.e., continence), urinary bother, sexual function, and sexual bother. We split the urinary bother scale into two sub-scales to distinguish incontinence-related bother from voiding dysfunction-related bother [16]. For each EPIC-50 sub-scale, a summary score was calculated and then transformed linearly to a 0-to-100 scale, with higher scores indicating better function and less bother.

### Demographic and lifestyle factors

Before RP, participants completed a baseline questionnaire including items on age, education, household income, race/ethnicity, insurance status, and marital status, as well as lifestyle factors, such as cigarette smoking history. Self-reported data on frequency of performing pelvic floor (i.e., Kegel) exercises after RP was ascertained on the follow-up questionnaires.

### BPH- and ED-related factors

We abstracted data on clinical characteristics from participants’ medical charts. These included comorbidities (Charlson Comorbidity Index), prostate cancer characteristics (pre-surgical clinical staging, prostate-specific antigen [PSA] concentration, and post-surgical pathological staging); pre-surgical urological conditions, including BPH (prostate size measured from the RP specimen, International Prostate Symptom Index [IPSS], and self-reported BPH medication use [overall, α-blocker use only, and 5α-reductase inhibitor use with or without α-blockers]); surgical characteristics taken from the operative note (blood loss during surgery, attempted number of neurovascular bundles preserved, type of surgical procedure, and bladder neck reconstruction), and sexual dysfunction therapies (pre- and post-surgical ED medication and device use).

### Statistical analysis

To begin to explore and display the distributions of urinary and sexual outcomes over time, we constructed boxplots with lines connecting the mean values from baseline through 12 months. This display is similar to figures presented in previous analyses of post-RP outcomes. Next, we investigated side effect trajectories by calculating the difference in each urinary and sexual outcome between baseline and 12 months for each participant and then by ranking participants according to their magnitude of change for each outcome (i.e., from the minimum to the maximum). To illustrate these trajectories, we selected a sample of participants (i.e., those ranked at the 5th, 25th, 50th, 75th, and 95th percentiles) for each outcome and plotted each participant’s change from baseline through 12 months, analogous to a selective “spaghetti plot”.

Given that some changes may be too small to be meaningful to patients, we next categorized both short- and long-term changes (from baseline to 5 weeks and 12 months, respectively) into clinically meaningful categories, using sub-scale-specific minimum clinically important difference (MCID) ranges reported by Skolarus and colleagues [17]. The upper bounds of these sub-scale-specific ranges were chosen to obtain conservative estimates: i.e., 9 point change in the urinary domain (7 for voiding dysfunction-related symptoms) and 12-point change in the sexual domain [17]. We used these sub-scale-specific values to create the following categories of change: improved beyond baseline (positive change greater than the MCID), maintained (within the positive to negative values of the MCID), and impaired below baseline (negative change greater than the MCID) [16, 18]. We also used the lower bounds of sub-scale-specific ranges to classify participants in sensitivity analyses.

Multinomial logistic regression was used to explore BPH- and ED-related factors associated with short- and long-term improvement beyond baseline and maintenance in urinary and sexual outcomes (in sub-scales with at least 10 men for stable estimation). Factors considered were individual measures of BPH (pre-surgical prostate size, IPSS score, and reported use of BPH medications, overall and separately by type), as well as a composite BPH outcome (prostate size ≥ 40 g [19], IPSS score ≥ 8 [20], or medication use), and measures of sexual dysfunction (pre- and post- surgical ED medication and device use). Sensitivity analyses were performed by: (1) adjusting for factors significantly associated with improvement or maintenance in at least one urinary or sexual outcome; (2) using the lower bounds of the EPIC-50 sub-scale-specific MCID ranges to classify participants; (3) excluding men with pre-surgical EPIC-50 scores too high to experience improvement (i.e., higher than the value obtained by subtracting the sub-scale specific MCID from 100); (4) restricting to men with complete data on specific urinary and sexual outcomes at baseline, 5 weeks and 12 months; and (5) repeating the analyses using the 6 month follow-up data.

## Results

### Participant characteristics

Of the 426 eligible participants who completed the baseline survey, 395 (92.7%) provided data on at least one EPIC-50 sub-scale at baseline, 5 weeks, or 12 months, and were included in the analysis. The majority of included participants (mean age = 60.7 years) were Caucasian (91.9%), had completed at least some college education (83.6%), earned ≥ $75,000 per year (61.4%), were married or living with a partner (82.5%), and had never smoked (62.5%, Table 1). Among participants with data on the Charlson Comorbidity Index (n = 167), 103 (61.7% of 103 and 26.2% of 394) had at least one comorbidity. Considering their prostate cancer-specific characteristics, most participants had clinical stage T1 (78.5%) and pathologic stage T3 (88.9%) disease, with a pre-surgical PSA concentration between 4 and 10 ng/mL. The majority (92.3%) of men underwent a minimally-invasive RP and 65.2% had a bilateral never-sparing procedure.

**Table 1:** Socio-demographic, lifestyle, and clinical characteristics of prostate cancer patients in the prostatectomy, incontinence and erectile dysfunction (PIE) study

	N	% or mean (SD)
*Socio-demographic and lifestyle factors*		
Age (years, mean, SD)	395	60.7 (6.9)
Caucasian (%)	385	91.9
Education (%)	385	
High school degree or less		16.4
Some college		30.9
College degree		23.9
Post graduate		28.8
Household income (%)	365	
< $50,000		18.3
$50,000—< $75,000		20.3
≥ $75,000		61.4
Married or living with a partner (%)	395	82.5
Smoking (%)	385	
Never smoker		62.5
Former smoker		32.7
Current smoker		6.8
Charlson Comorbidity Index (%)	394	
No comorbidities		16.2
Any comorbidities		26.2
Missing		57.6
*Prostate cancer- and surgery-related factors*		
Clinical T1 stage (%)	381	78.5
Pathological stage (%)	389	
T2		11.1
T3		88.9
Pre-surgical prostate-specific antigen concentration (ng/mL, mean, SD)	393	6.3 (4.6)
Blood loss during surgery (mL, mean, SD)	358	246.3 (203.3)
Neurovascular bundle preservation (%)	351	
Non-nerve sparing		19.4
Unilateral neurovascular bundle spared		15.4
Bilateral nerve sparing		65.2
Surgical procedure (%)	365	
Minimally-invasive (robotic and laparoscopic)		92.3
Open		7.7
Bladder reconstruction	364	53.0
*BPH-related factors*		
Prostate size (grams, mean, SD)	288	43.5 (16.6)
IPSS score at baseline (mean, SD)	249	8.6 (6.7)
BPH medication use at baseline (%)	395	13.2
α-blocker use		10.4
5α-reductase inhibitor (and α-blocker) use		2.8
*Urinary and sexual function-related factors*		
Reported Kegel exercises		
5 weeks post-surgery	358	67.3
12 months post-surgery	332	31.3
ED medication or device use (%)		
Baseline	365	4.8
5 weeks post-surgery	365	30.1
12 months post-surgery	336	18.2

*BPH* benign prostatic hyperplasia, *ED* erectile dysfunction, *IPSS* International Prostate Symptom Score, *SD* standard deviation

### Changes in urinary and sexual outcomes

**Fig. 1:**
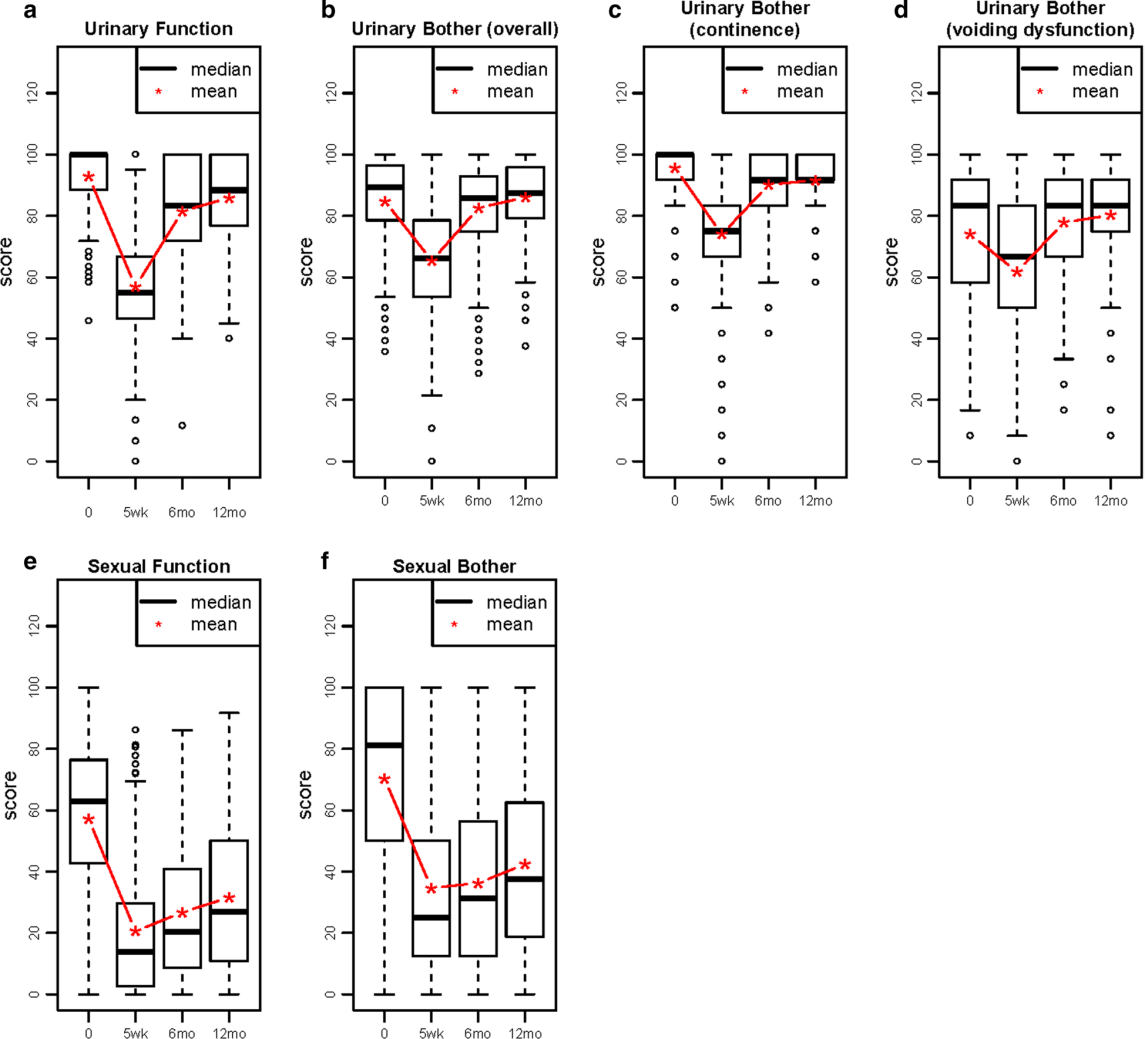
Changes in Urinary and Sexual Outcomes (mean values) Assessed by the Expanded Prostate Cancer Index Composite (EPIC)-50 among Men in the Prostatectomy, Incontinence and Erectile Function (PIE) Study, between Baseline and 12 Months Post-Radical Prostatectomy

With respect to urinary incontinence, most men had high function (mean = 93.1) and bother scores (mean = 95.8, i.e., good function and not much bother) at baseline. Scores for voiding dysfunction-related bother were also high (mean = 74.5), but lower than for incontinence-related bother. With respect to sexual outcomes, baseline levels were lower than for urinary outcomes, and were also lower for sexual function (mean = 56.2) than for bother (mean = 68.9), indicating worse sexual function but not as much bother (Fig. 1a–e). Five weeks after surgery, mean levels of each of these outcomes were markedly decreased. For urinary incontinence-related outcomes, levels recovered to near, but below, baseline 6 months post-surgery and then slowed to a plateau by 12 months. In contrast, for voiding dysfunction-related bother, levels recovered to above baseline 6 months post-surgery and then remained relatively constant through 12 months. Finally, for sexual-related outcomes, mean levels remained well below baseline but continued to improve gradually through 12 months post-surgery.

**Fig. 2:**
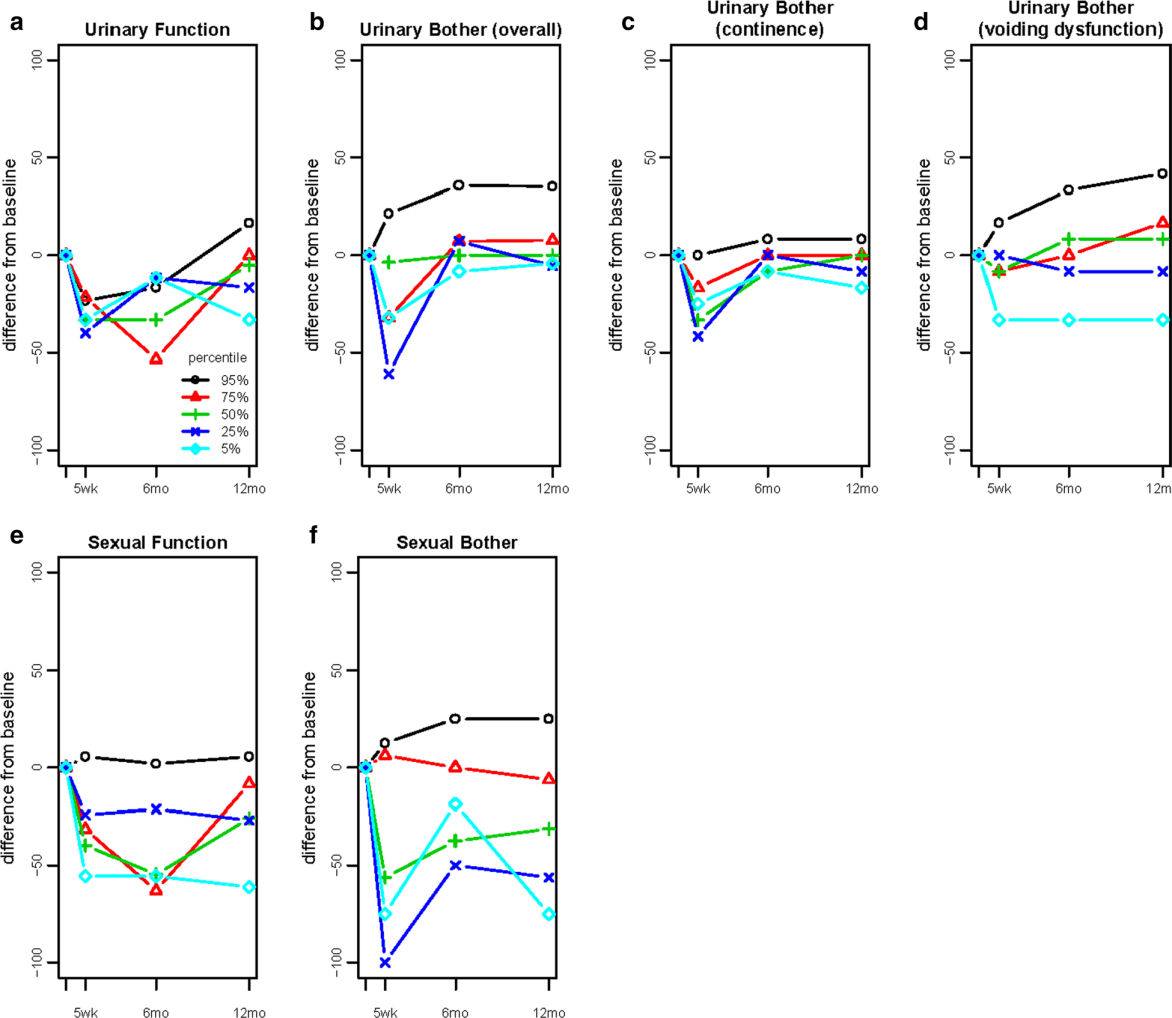
Individual Trajectories of Urinary and Sexual Outcomes Assessed by the Expanded Prostate Cancer Index Composite (EPIC)-50 among Men in the Prostatectomy, Incontinence and Erectile Function (PIE) Study from Baseline through 12 Months Post-Radical Prostatectomy (The trajectories for five different individuals per domain are presented in each graph. These individuals were selected by subtracting each participant’s 12 month follow-up values in each EPIC-50 domain from their baseline values and then by ranking these differences. The outcome trajectories for participants ranked at the 5^th^, 25^th^, 50^th^, 75^th^, and 95^th^ percentiles of change for each domain are presented to illustrate the range of trajectories experienced by participants. In each domain, participants at the 95^th^ percentile experienced improvement)

When individual trajectories of change were examined, a generally similar impression of symptom change was obtained, particularly when viewing the median (50^th^) percentile trajectories (Fig. 2a–e). However, these displays also highlighted improved (beyond baseline) or maintained outcomes in all domains over time. Improvement was greatest for voiding dysfunction-related bother (22.1% of men at 5 weeks; 50.9% at 12 months), followed by sexual bother (7.3% at 5 weeks; 10.6% at 12 months), and urinary function (9.8% at 12 months, Tables 2 and 3). Lesser proportions of men experienced improvement in incontinence-related bother and sexual function (< 5%). Of note, improvement beyond baseline was observed even as early as 5 weeks post-surgery, particularly for voiding dysfunction-related and sexual bother.

**Table 2:** Factors Associated with urinary outcome trajectories assessed by the expanded prostate cancer index composite (EPIC)-50 among men in the prostatectomy, incontinence and erectile dysfunction (PIE) study

	All participants n (%)	BPH-related factors								ED-related factors			
		Prostate size		IPSS score		BPH medication use at baseline		BPH composite measure		ED medication use at baseline		ED medication or device use at follow-up	
		< 40 g	≥ 40 g	< 8	≥ 8	No	Yes	No BPH	Any BPH	No	Yes	No	Yes
Urinary function (continence) 5 weeks post-surgery (n = 377)													
Impaired													
N	346 (91.8)												
OR 95% CI													
Maintained													
N	28 (7.4)												
OR 95% CI													
Improved													
N	3 (0.8)												
OR 95% CI													
Urinary function (continence) 12 months post-surgery (n = 347)													
Impaired													
N	145 (41.8)	57	51	53	46	129	16	63	82	137	8	46	94
OR 95% CI		Reference		Reference		Reference		Reference		Reference		Reference	
Maintained													
N	168 (48.4)	52	71	58	51	148	20	64	104	163	5	59	102
OR 95% CI		1.5 (0.9 to 2.6)		1.0 (0.6 to 1.7)		1.1 (0.5 to 2.2)		1.2 (0.8 to 2.0)		0.5 (0.2 to 1.6)		0.8 (0.5 to 1.4)	
Improved													
N	34 (9.8)	11	17	6	11	28	6	11	23	31	3	13	18
OR 95% CI		1.7 (0.6 to 4.0)		2.1 (0.7 to 6.2)		1.7 (0.6 to 4.8)		1.6 (0.7 to 3.5)		1.7 (0.4 to 6.6)		0.7 (0.3 to 1.5)	
Incontinence-related bother 5 weeks post-surgery (n = 379)													
Impaired													
N	266 (70.3)												
OR 95% CI													
Maintained													
N	110 (29.0)												
OR 95% CI													
Improved													
N	3 (0.8)												
OR 95% CI													
Incontinence-related bother 12 months post-surgery (n = 348)													
Impaired													
N	53 (15.2)	16	22	25	14	47	6	23	30	50	3	18	33
OR 95% CI		Reference		Reference		Reference		Reference		Reference		Reference	
Maintained													
N	282 (81.0)	103	109	91	87	251	31	113	169	269	13	97	174
OR 95% CI		0.8 (0.4 to 1.5)		1.7 (0.8 to 3.5)		1 (0.4 to 2.4)		1.1 (0.6 to 2.1)		0.8 (0.2 to 2.9)		1.0 (0.5 to 1.8)	
Improved													
N	13 (3.7)	5	7	2	6	9	4	3	10	13	0	6	6
OR 95% CI		1.0 (0.3 to 3.8)		5.4 (1.0 to 30.2)		3.5 (0.8 to 15)		2.6 (0.6 to 10.4)		NE		0.5 (0.2 to 1.9)	
Voiding dysfunction-related bother 5 weeks post-surgery (n = 380)													
Impaired													
N	238 (62.6)	93	89	91	54	212	26	109	129	226	12	126	99
OR 95% CI		Reference		Reference		Reference		Reference		Reference		Reference	
Maintained													
N	58 (15.3)	21	18	23	15	54	4	27	31	55	3	22	33
OR 95% CI		0.9 (0.4 to 1.8)		1.1 (0.5 to 2.3)		0.6 (0.2 to 1.8)		1.0 (0.5 to 1.7)		1.0 (0.3 to 3.8)		1.9 (1.0 to 3.5)	
Improved													
N	84 (22.1)	20	41	14	46	63	21	15	69	80	4	38	40
OR 95% CI		2.1 (1.2 to 3.9)		5.5 (2.8 to 11.0)		2.7 (1.4 to 5.2)		3.9 (2.1 to 7.2)		0.9 (0.3 to 3.0)		1.3 (0.8 to 2.2)	
Voiding dysfunction-related bother 12 months post-surgery (n = 348)													
Impaired													
N	91 (26.1)	38	32	36	20	84	7	49	42	87	4	32	57
OR 95% CI		Reference		Reference		Reference		Reference		Reference		Reference	
Maintained													
N	80 (23.0)	37	26	35	13	75	5	44	36	76	4	22	53
OR 95% CI		0.8 (0.4 to 1.7)		0.7 (0.3 to 1.5)		0.8 (0.2 to 2.6)		1.0 (0.5 to 1.7)		1.1 (0.3 to 4.7)		1.4 (0.7 to 2.6)	
Improved													
N	177 (50.9)	49	80	47	74	148	29	46	131	169	8	67	103
OR 95% CI		1.9 (1.1 to 3.5)		2.8 (1.5 to 5.5)		2.4 (1.0 to 5.6)		3.3 (2.0 to 5.7)		1.0 (0.3 to 3.5)		0.9 (0.5 to 1.5)	

*BPH* benign prostatic hyperplasia, *CI* confidence interval, *ED* erectile dysfunction, *IPSS* International Prostate Symptom Score, *OR* odds ratio, *NE* not estimable

EPIC-50 measured urinary and sexual outcome trajectories were defined as impaired (negative change greater than the upper bound of minimally important difference), maintained, and improved (positive change greater than the upper bound of minimally important difference). Urinary function, incontinence-related bother: impaired (change < 9); maintained (− 9 ≤ change ≤ 9); improved (change ≥ 9). Voiding dysfunction-related bother: impaired (change < 7); maintained (− 7 ≤ change ≤ 7); improved (change ≥ 7)

**Table 3:** Factors associated with sexual outcome trajectories assessed by the expanded prostate cancer index composite (EPIC)-50 among men in the prostatectomy, incontinence and erectile dysfunction (PIE) study

	All participants n (%)	BPH-related factors								ED -elated factors			
		Prostate size		IPSS score		BPH medication use at baseline		BPH composite measure		ED medication use at baseline ED medication use at baseline		ED medication or device use at follow-up	
		< 40 g	≥ 40 g	< 8	≥ 8	No	Yes	No BPH	Any BPH	No	Yes	No	Yes
Sexual function 5 weeks post-surgery (n = 357)													
Impaired													
N	262 (79.0)												
OR 95% CI													
Maintained													
N	69 (19.3)												
OR 95% CI													
Improved													
N	6 (1.7)												
OR 95% CI													
Sexual function 12 months post-surgery (n = 332)													
Impaired													
N	229 (69.0)	76	96	73	73	205	24	91	138	217	12	70	154
OR 95% CI		Reference		Reference		Reference		Reference		Reference		Reference	
Maintained													
N	92 (27.7)	39	28	35	28	80	12	40	52	88	4	40	48
OR 95% CI		0.6 (0.3 to 1.0)		0.8 (0.4 to 1.4)		1.3 (0.6 to 2.7)		0.8 (0.5 to 1.4)		0.8 (0.3 to 2.6)		0.5 (0.3 to 0.9)	
Improved													
N	11 (3.3)	3	6	4	4	8	3	2	9	11	0	2	5
OR 95% CI		1.6 (0.4 to 6.5)		1.0 (0.2 to 4.2)		3.2 (0.8 to 13)		3.0 (0.6 to 14)		NE		1.1 (0.2 to 6.0)	
Sexual bother 5 weeks post-surgery (n = 354)													
Impaired													
N	259 (73.2)	94	96	86	77	232	27	110	149	243	16	117	131
OR 95% CI		Reference		Reference		Reference		Reference		Reference		Reference	
Maintained													
N	69 (19.5)	27	27	27	22	61	8	30	39	69	0	36	24
OR 95% CI		1.0 (0.5 to 1.8)		0.9 (0.5 to 1.7)		1.1 (0.5 to 2.6)		1.0 (0.6 to 1.6)		NE		0.6 (0.3 to 1.1)	
Improved													
N	26 (7.3)	6	11	8	11	16	10	3	23	24	2	16	10
OR 95% CI		1.8 (0.6 to 5.1)		1.5 (0.6 to 4.0)		5.4 (2.2 to 13.0)		5.7 (1.7 to 19.3)		1.3 (0.3 to 5.8)		0.6 (0.2 to 1.3)	
Sexual bother 12 months post-surgery (n = 329)													
Impaired													
N	229 (69.6)	78	89	81	67	206	23	98	131	216	13	68	157
OR 95% CI		Reference		Reference		Reference		Reference		Reference		Reference	
Maintained													
N	65 (19.8)	28	23	22	19	56	9	28	37	62	3	27	34
OR 95% CI		0.7 (0.4 to 1.4)		1.0 (0.5 to 2.1)		1.4 (0.6 to 3.3)		1.0 (0.6 to 1.7)		0.8 (0.2 to 2.9)		0.5 (0.3 to 1.0)	
Improved													
N	35 (10.6)	8	19	11	15	30	5	7	28	35	0	13	16
OR 95% CI		2.1 (0.9 to 5.0)		1.6 (0.7 to 3.8)		1.5 (0.5 to 4.2)		3.0 (1.2 to 7.1)		NE		0.5 (0.2 to 1.2)	

*BPH* benign prostatic hyperplasia, *CI* confidence interval, *ED* erectile dysfunction, *IPSS* International Prostate Symptom Score, *OR* odds ratio, *NE* not estimable

EPIC-50 measured urinary and sexual outcome trajectories were defined as impaired (negative change greater than the upper bound of minimally important difference), maintained, and improved (positive change greater than the upper bound of minimally important difference). Sexual function, sexual bother: impaired (change < 12); maintained (-12 ≤ change ≤ 12); improved (change ≥ 12)

### Factors associated with improvement beyond baseline in urinary and sexual outcomes

No significant associations were observed for BPH- or ED-related factors with improved or maintained urinary function or incontinence-related bother after surgery (only evaluable at 12 months, Table 2 and Additional file 1: Table 1). In contrast, each of the individual measures of BPH, as well as the BPH composite measure, were associated with improved voiding dysfunction-related bother at both 5 weeks and 12 months post-surgery (5 weeks: odds ratio [OR] = 3.9, 95% confidence interval [CI]: 2.1–7.2; 12 months: OR = 3.3, 95% CI: 2.0–5.7 for the BPH composite measure). No associations were observed for ED medication or device use at baseline or follow-up.

With respect to sexual outcomes, no significant associations were observed for BPH- or ED-related factors with improved or maintained sexual function. In contrast, the BPH composite measure was associated with improved sexual bother at both 5 weeks (OR = 5.7, 95% CI:1.7–19.3) and 12 months post-surgery (OR = 3.0, 95% CI: 1.2–7.1, Table 3 and Additional file 1: Table 2). Positive associations were also observed for several of the individual measures of BPH, including α-blocker use (OR = 4.4, 95% CI: 1.1–18.0) with improvement in sexual function at 12 months post-surgery, and BPH medication use (OR = 5.4, 95% CI: 2.2–13.0) and α-blocker use (OR = 5.6, 95% CI: 2.2–14.6) with improvement in sexual bother at 5 weeks. Finally, an inverse association was observed for ED medication or device use during follow-up with maintained sexual function (OR = 0.5, 95% CI: 0.3–0.9) and bother (OR = 0.5, 95% CI: 0.3–1.0) compared to impaired function and bother 12 months post-surgery. This is likely due to confounding by indication, whereby men who experienced large declines in sexual function may have been more likely to use ED medications or devices after surgery.

Similar results were observed in sensitivity analyses: (1) adjusting for age, smoking status, living arrangement, and neurovascular bundle preservation; (2) using the lower bounds of the EPIC-50 sub-scale-specific MCID ranges; (3) excluding men with high pre-surgical scores in each EPIC-50 sub-scale; (4) restricting to men with complete outcome data at baseline, 5 weeks and 12 months; and (5) examining the 6-month follow-up data (results not shown).

## Discussion

Similar to previous studies, our study demonstrated sharp declines in mean urinary and sexual outcomes 5 weeks post-RP, followed by recovery to near, but below, baseline values for urinary incontinence-related outcomes, and to improved, but well below, baseline values for sexual outcomes. However, by examining individual participant trajectories, we also identified several groups of men who experienced improvement beyond their baseline values in each urinary and sexual outcome. Notably, a considerable proportion of men experienced immediate and long-term improvement in voiding dysfunction-related bother, and a small proportion experienced long-term improvement in urinary function (continence) and sexual bother. To our knowledge, improvement in outcomes besides voiding dysfunction has not previously been documented. Additionally, we found that pre-surgical BPH was associated with improvement beyond baseline in both voiding dysfunction and sexual bother, strengthening the evidence in national urologic oncology guidelines to recommend surgical treatment for appropriate prostate cancer patients with BPH.

Our finding of clinically meaningful improvement in urinary continence among a small proportion of men treated for prostate cancer by RP was unexpected and differs from most previously published findings [10, 21, 22]. One possible explanation for this finding may be surgical elimination of bladder outlet resistance by RP. Studies of bladder outlet resistance have demonstrated that the bladder detrusor muscle undergoes structural and functional changes, with initial hypertrophy, then compensation, and then decompensation [23–25]. The bladders of men with BPH who undergo RP for prostate cancer may lie anywhere along this spectrum. Men in an initial hypertrophic stage may have some degree of urge-related urinary incontinence, which may be relieved by bladder remodelling following surgical elimination of outlet resistance by RP. Likewise, men in a late decompensated stage may have some degree of stress-related urinary incontinence owing to chronic subclinical urinary retention, which may also be relieved by RP.

An additional unexpected observation was our finding that a small proportion of men who underwent RP experienced improvements beyond baseline in sexual outcomes, particularly long-term sexual bother, independent of ED therapy. Although resolution of neuropraxia might explain recovery in these outcomes to baseline levels, we believe it is unlikely to explain improvements beyond baseline. Another possible explanation is improved communication surrounding sexual function. Prostate cancer treatment and its known sexual side effects may be a launching point for men to have an open conversation with their providers and partners about their sexual function. Previous studies have demonstrated that spousal communication is a key factor in healthy sexual function recovery after prostate cancer treatment [26]. For many men, prostate cancer treatment discussions may prompt discussions regarding sexual function that have not been addressed in the past. The mechanisms through which open communication may improve post-RP sexual outcomes might be similar to those in studies of premenopausal women with dyspareunia, which found that open communication between partners was critical for improving sexual function and distress [27].

Another possible explanation for improvement in sexual bother could be relief of sexual bother related to voiding dysfunction post-RP. This hypothesis is supported by growing evidence that BPH and ED may be caused by common biologic mechanisms [28]. As such, improvements in BPH-related symptoms from RP may also result in improvements in sexual outcomes. Alternatively, certain BPH-related medications are known to be associated with decreased sexual function [29]. Therefore, discontinuation of these drugs may contribute to improvement following RP, as was observed for men taking α-blockers pre-RP in our sample. In support of both of these mechanisms, our data demonstrated a higher odds of improved sexual bother associated with the BPH composite index. Finally, it is also possible that RP may relieve sexual pain in men with pre-surgical chronic prostatitis/chronic pelvic pain syndrome, thereby contributing to improvements in sexual bother. Although each of these explanations is speculative, we believe they warrant further study for their possible, eventual guidance for prostate cancer therapeutic decision-making and overall patient counseling.

Strengths of this study include its prospective design, large sample size, frequent follow-up of participants over the one-year study period, and use of validated outcome measures designed specifically for prostate cancer survivors. Additionally, use of the EPIC-50 rather than the shorter EPIC-26 allowed us to gain a more comprehensive understanding of both urinary and sexual function and bother; and our unique statistical analysis allowed us to explore the full range of outcome trajectories rather than just mean outcome levels. However, it is also important to note the limitations of this analysis. Specifically, information on co-existing urological conditions was not collected systematically on all participants, but was only available from participants ‘ medical records, which may not have had complete information on these conditions. Additionally, data were not collected between 5 weeks and 6 months (e.g., 3 months) to allow us to identify when some outcomes returned to baseline levels.

## Conclusions

We observed improvements in urinary and sexual outcomes among non-trivial proportions of men who underwent RP. Although reasons for improvement in urinary function (continence) and sexual bother are unclear, improvement in voiding dysfunction-related bother likely relates to relief of BPH symptoms by RP, as prostatectomy is a known, effective therapy for severe BPH. Therefore, our findings provide additional support for considering baseline BPH symptoms when selecting the best therapy for early-stage prostate cancer.

## Supplementary Information

**Additional file 1: Supplementary Table 1**. Association between Baseline BPH Medication Use with Urinary Outcome Trajectories Assessed by the Expanded Prostate Cancer Index Composite (EPIC)-50 among Men in the Prostatectomy, Incontinence and Erectile Dysfunction (PIE) Study. **Supplementary Table 2**. Association between Baseline BPH Medication Use with Sexual Outcome Trajectories Assessed by the Expanded Prostate Cancer Index Composite (EPIC)-50 among Men in the Prostatectomy, Incontinence and Erectile Dysfunction (PIE) Study.

## Data Availability

The dataset supporting the conclusion of this article is available upon requst.
