# Supplementary material for: One-year urinary and sexual outcome trajectories among prostate cancer patients treated by radical prostatectomy: a prospective study
Source: BMC Urol. 2021 May 17;21:81. doi: 10.1186/s12894-021-00845-0 (PMC8130427; doi:10.1186/s12894-021-00845-0)
Supplement: Supplementary file 1 — Additional file 1: Supplementary Table 1. Association between Baseline BPH Medication Use with Urinary Outcome Trajectories Assessed by the Expanded Prostate Cancer Index Composite (EPIC)-50 among Men in the Prostatectomy, Incontinence and Erectile Dysfunction (PIE) Study. Supplementary Table 2. Association between Baseline BPH Medication Use with Sexual Outcome Trajectories Assessed by the Expanded Prostate Cancer Index Composite (EPIC)-50 among Men in the Prostatectomy, Incontinence and Erectile Dysfunction (PIE) Study. [file 12894_2021_845_MOESM1_ESM.docx]

| Supplementary Table 1. Association between Baseline BPH Medication Use with Urinary Outcome Trajectories Assessed by the Expanded Prostate Cancer Index Composite (EPIC)-50 among Men in the Prostatectomy, Incontinence and Erectile Dysfunction (PIE) Study | | | | | | | | | | | | | | | | | |
| --- | --- | --- | --- | --- | --- | --- | --- | --- | --- | --- | --- | --- | --- | --- | --- | --- | --- |
|  | | | | | |  |  | |  | BPH-medication use at baseline | | | | | | | |
|  | | | | | |  | All participants  n (%) | |  | α-blockers | | |  | 5α-reductase inhibitors (and α-blockers) | | |  |
|  | | | | | |  |  |  |  | No | | Yes |  | No | | Yes |  |
| Urinary function (continence) 5 weeks post-surgery (n=377) | | | | | | | | | | | | | | | | | |
| Impaired |  | | | N | |  | 346 (91.8) | |  |  | |  |  |  | |  |  |
|  |  | | | OR 95% CI | |  |  | |  |  | | |  |  | | |  |
| Maintained |  | | | N | |  | 28 (7.4) | |  |  | |  |  |  | |  |  |
|  |  | | | OR 95% CI | |  |  | |  |  | | |  |  | | |  |
| Improved |  | | | N | |  | 3 (0.8) | |  |  | |  |  |  | |  |  |
|  |  | | | OR 95% CI | |  |  | |  |  | | |  |  | | |  |
| Urinary function (continence) 12 months post-surgery (n=347) | | | | | | | | | | | | | | | | | |
| Impaired |  | | | N | |  | 145 (41.8) | |  | *132* | *13* | |  | *142* | *3* | |  |
|  |  | | | OR 95% CI | |  |  | |  | Reference | | |  | Reference | | |  |
| Maintained |  | | | N | |  | 168 (48.4) | |  | *152* | *16* | |  | *164* | *4* | |  |
|  |  | | | OR 95% CI | |  |  | |  | 1.1 (0.5 to 2.3) | | |  | 1.2 (0.3 to 5.2) | | |  |
| Improved |  | | | N | |  | 34 (9.8) | |  | *30* | *4* | |  | *32* | *2* | |  |
|  |  | | | OR 95% CI | |  |  | |  | 1.4 (0.4 to 4.4) | | |  | 3.0 (0.5 to 18.4) | | |  |
| Incontinence-related bother 5 weeks post-surgery (n=379) | | | | | | | | | | | | | | | | | |
| Impaired | |  | N | |  | | | 266 (70.3) |  |  | |  |  |  | |  |  |
|  |  |  | OR 95% CI | |  | | |  |  |  | |  |  |  | |  |  |
| Maintained | |  | N | |  | | | 110 (29.0) |  |  | |  |  |  | |  |  |
|  |  |  | OR 95% CI | |  | | |  |  |  | |  |  |  | |  |  |
| Improved | |  | N | |  | | | 3 (0.8) |  |  | |  |  |  | |  |  |
|  |  |  | OR 95% CI | |  | | |  |  |  | |  |  |  | |  |  |
| Incontinence-related bother 12 months post-surgery (n=348) | | | | | | | | | | | | | | | | | |
| Impaired |  | | | N | |  | 53 (15.2) | |  | *47* | | *6* |  | *53* | | *0* |  |
|  |  | | | OR 95% CI | |  |  | |  | Reference | | |  | Reference | | |  |
| Maintained |  | | | N | |  | 282 (81.0) | |  | *260* | | *22* |  | *273* | | *9* |  |
|  |  | | | OR 95% CI | |  |  | |  | 0.7 (0.3 to 1.7) | | |  | NE | | |  |
| Improved |  | | | N | |  | 13 (3.7) | |  | *9* | | *4* |  | *13* | | *0* |  |
|  |  | | | OR 95% CI | |  |  | |  | 3.5 (0.8 to 14.9) | | |  | NE | | |  |
| Voiding dysfunction-related bother 5 weeks post-surgery (n=380) | | | | | | | | | | | | | | | | | |
| Impaired |  | | | N | |  | 238 (62.6) | |  | *219* | | *19* |  | *231* | | *7* |  |
|  |  | | | OR 95% CI | |  |  | |  | Reference | | |  | Reference | | |  |
| Maintained |  | | | N | |  | 58 (15.3) | |  | *54* | | *4* |  | *58* | | *0* |  |
|  |  | | | OR 95% CI | |  |  | |  | 0.9 (0.3 to 2.6) | | |  | NE | | |  |
| Improved |  | | | N | |  | 84 (22.1) | |  | *67* | | *17* |  | *80* | | *4* |  |
|  |  | | | OR 95% CI | |  |  | |  | 2.9 (1.4 to 5.9) | | |  | 1.7 (0.8 to 5.8) | | |  |
| Voiding dysfunction-related bother 12 months post-surgery (n=348) | | | | | | | | | | | | | | | | | |
| Impaired |  | | | N | |  | 91 (26.1) | |  | *85* | | *6* |  | *90* | | *1* |  |
|  |  | | | OR 95% CI | |  |  | |  | Reference | | |  | Reference | | |  |
| Maintained |  | | | N | |  | 80 (23.0) | |  | *75* | | *5* |  | *80* | | *0* |  |
|  |  | | | OR 95% CI | |  |  | |  | 0.9 (0.3 to 3.2) | | |  | NE | | |  |
| Improved |  | | | N | |  | 177 (50.9) | |  | *156* | | *21* |  | *169* | | *8* |  |
|  |  | | | OR 95% CI | |  |  | |  | 1.9 (0.7 to 4.9) | | |  | 4.3 (0.5 to 34.7) | | |  |
| BPH=benign prostatic hyperplasia; CI=confidence interval; ED=erectile dysfunction; IPSS=International Prostate Symptom Score; OR=odds ratio; NE=not estimable.  EPIC-50 measured urinary and sexual outcome trajectories were defined as impaired (negative change greater than the upper bound of minimally important difference), maintained, and improved (positive change greater than the upper bound of minimally important difference). Urinary function, incontinence-related bother: impaired (change < 9); maintained (-9 ≤ change ≤ 9); improved (change ≥ 9). Voiding dysfunction-related bother: impaired (change < 7); maintained (-7 ≤ change ≤ 7); improved (change ≥ 7). | | | | | | | | | | | | | | | | | |

| Supplementary Table 2. Association between Baseline BPH Medication Use with Sexual Outcome Trajectories Assessed by the Expanded Prostate Cancer Index Composite (EPIC)-50 among Men in the Prostatectomy, Incontinence and Erectile Dysfunction (PIE) Study | | | | | | | | | | | | | | | | | |
| --- | --- | --- | --- | --- | --- | --- | --- | --- | --- | --- | --- | --- | --- | --- | --- | --- | --- |
|  | | | | | |  |  | |  | BPH-medication use at baseline | | | | | | | |
|  | | | | | |  | All participants  n (%) | |  | α-blockers | | |  | 5α-reductase inhibitors (and α-blockers) | | |  |
|  | | | | | |  |  |  |  | No | | Yes |  | No | | Yes |  |
| Sexual function 5 weeks post-surgery (n=357) | | | | | | | | | | | | | | | | | |
| Impaired |  | | | N | |  | 262 (79.0) | |  |  | |  |  |  | |  |  |
|  |  | | | OR 95% CI | |  |  | |  |  | | |  |  | | |  |
| Maintained |  | | | N | |  | 69 (19.3) | |  |  | |  |  |  | |  |  |
|  |  | | | OR 95% CI | |  |  | |  |  | | |  |  | | |  |
| Improved |  | | | N | |  | 6 (1.7) | |  |  | |  |  |  | |  |  |
|  |  | | | OR 95% CI | |  |  | |  |  | | |  |  | | |  |
| Sexual function 12 months post-surgery (n=332) | | | | | | | | | | | | | | | | | |
| Impaired |  | | | N | |  | 229 (69.0) | |  | *211* | *18* | |  | *223* | *6* | |  |
|  |  | | | OR 95% CI | |  |  | |  | Reference | | |  | Reference | | |  |
| Maintained |  | | | N | |  | 92 (27.7) | |  | *82* | *10* | |  | *90* | *2* | |  |
|  |  | | | OR 95% CI | |  |  | |  | 1.4 (0.6 to 3.2) | | |  | 0.8 (0.2 to 4.2) | | |  |
| Improved |  | | | N | |  | 11 (3.3) | |  | *8* | *3* | |  | *11* | *0* | |  |
|  |  | | | OR 95% CI | |  |  | |  | 4.4 (1.1 to 18.0) | | |  | NE | | |  |
| Sexual bother 5 weeks post-surgery (n=354) | | | | | | | | | | | | | | | | | |
| Impaired | |  | N | |  | | | 259 (73.2) |  | *240* | | *19* |  | *251* | | *8* |  |
|  |  |  | OR 95% CI | |  | | |  |  | Reference | | |  | Reference | | |  |
| Maintained | |  | N | |  | | | 69 (19.5) |  | *61* | | *8* |  | *69* | | *0* |  |
|  |  |  | OR 95% CI | |  | | |  |  | 1.6 (0.7 to 4.0) | | |  | NE | | |  |
| Improved | |  | N | |  | | | 26 (7.3) |  | *18* | | *8* |  | *24* | | *2* |  |
|  |  |  | OR 95% CI | |  | | |  |  | 5.6 (2.2 to 14.6) | | |  | 2.6 (0.5 to 13.0) | | |  |
| Sexual bother 12 months post-surgery (n=329) | | | | | | | | | | | | | | | | | |
| Impaired |  | | | N | |  | 229 (69.6) | |  | *211* | | *18* |  | *224* | | *5* |  |
|  |  | | | OR 95% CI | |  |  | |  | Reference | | |  | Reference | | |  |
| Maintained |  | | | N | |  | 65 (19.8) | |  | *56* | | *9* |  | *65* | | *0* |  |
|  |  | | | OR 95% CI | |  |  | |  | 1.9 (0.8 to 4.4) | | |  | NE | | |  |
| Improved |  | | | N | |  | 35 (10.6) | |  | *31* | | *4* |  | *34* | | *1* |  |
|  |  | | | OR 95% CI | |  |  | |  | 1.5 (0.5 to 4.8) | | |  | 1.3 (0.1 to 11.6) | | |  |
| BPH=benign prostatic hyperplasia; CI=confidence interval; ED=erectile dysfunction; IPSS=International Prostate Symptom Score; OR=odds ratio; NE=not estimable.  EPIC-50 measured urinary and sexual outcome trajectories were defined as impaired (negative change greater than the upper bound of minimally important difference), maintained, and improved (positive change greater than the upper bound of minimally important difference). Urinary function, incontinence-related bother: impaired (change < 9); maintained (-9 ≤ change ≤ 9); improved (change ≥ 9). Voiding dysfunction-related bother: impaired (change < 7); maintained (-7 ≤ change ≤ 7); improved (change ≥ 7). | | | | | | | | | | | | | | | | | |
